# Expression patterns of major genes in fatty acid synthesis, inflammation, oxidative stress pathways from colostrum to milk in Damascus goats

**DOI:** 10.1038/s41598-021-88976-0

**Published:** 2021-05-03

**Authors:** Akın Yakan, Hüseyin Özkan, Baran Çamdeviren, Ufuk Kaya, İrem Karaaslan, Sevda Dalkiran

**Affiliations:** 1 Department of Genetics, Faculty of Veterinary Medicine, Hatay Mustafa Kemal University, 31060 Hatay, Turkey; 2 Department of Molecu1ar Biochemistry and Genetics, Institute of Health Sciences, Hatay Mustafa Kemal University, 31060 Hatay, Turkey; 3 Department of Biostatistics, Faculty of Veterinary Medicine, Hatay Mustafa Kemal University, 31060 Hatay, Turkey; 4 Technology and Research and Development Center (MARGEM), Hatay Mustafa Kemal University, 31060 Hatay, Turkey

**Keywords:** Genetics, Molecular biology, Physiology, Zoology, Biomarkers

## Abstract

The molecular regulation of milk secretion and quality in the transition period from colostrum to milk in goats is largely unknown. In the present study, mammary gland secretion of goats was collected in 0th, 4th, 7th, 14th and 28th days after parturition. In addition to composition and fatty acid profile of colostrum or milk, *FASN*, *SCD*, *ACACA*, *COX-2*, *NRF2*, *TLR2*, *NF-kB*, *LTF* and *PTX3* genes expression patterns were determined from milk somatic cells. While somatic cell count (SCC), malondialdehyde (MDA), fat, fat-free dry matter, protein and lactose were highest as expression levels of the oxidative and inflammatory genes, freezing point and electrical conductivity were lowest in colostrum. With the continuation of lactation, most of the fatty acids, n3 ratio, and odour index increased but C14:0 and C16:0 decreased. While *FASN* was upregulated almost threefolds in 14th day, *ACACA* was upregulated more than fivefolds in 7th and 14th days. Separately, the major genes in fatty acid synthesis, inflammation and oxidative stress were significantly associated with each other due to being positively correlated. MDA was positively correlated with SCC and some of the genes related inflammation and oxidative stress. Furthermore, significant negative correlations were determined between SCC and fatty acid synthesis related genes. With this study, transition period of mammary secretion was particularly clarified at the molecular levels in Damascus goats.

## Introduction

The world human population is expected to be around 10 billion by 2050^1^. Animal production targets are rising in response to the increasing world population^2^. Goat is an important farm animal for the production of dairy products, as well as for the supply of healthy, high-quality and valuable food, especially for the nutrition of children, the elderly and those with food allergies^3,4^. With the increasing demand for goat milk in recent years, goat breeding has gained importance and the amount of milk production has increased with the number of goats^5,6^.

Milk, an important animal product, is a vital biological secretion that begins with the birth. Colostrum which is the first secretion of mammary tissue has quietly different from mature milk^7^. Besides its immunological function, colostrum is pivotal for the initial intake of nutrients in terms of the survival and sustainable health of the newborn^8^. Regulation of mammary secretion is controlled by local and systemic factors. Therefore, colostrum and milk components are regulated by different mechanisms^8^.

Goat colostrum is richer in somatic cells, fat, protein, fat-free dry matter (FFDM), lactose and immunoglobulins^8,9^. The considered changes occur in composition and properties during the transition from colostrum to mature milk. It has been reported that goat colostrum turns into milk in 4–5 days after birth goats^9,10^. On the other hand, transition to mature milk secretion is a process rather than straight conversion.

As a well-known fact, milk traits are affected by genetic and environmental factors such as breed type, age, and parity. In addition, unlike other farm animals, the milk secretion type of goats is apocrine^3^. Due to the type of secretion, somatic cell count may be high in goat milk^11^. Colostrum also contains quite high SCC compared to mature milk as well as its difference in composition^8,12^. On the other hand, molecular activity in milk somatic cells may reflect mammary physiology^11,13^. In this way, it is possible to determine molecular activity of mammary gland without the need for invasive methods such as biopsy. It is known that invasive methods have possibility of mammary fistula and mastitis^11,14^. Several studies have deepened to improve the molecular basis of milk secretion in cattle, however, what is known about the molecular basis in the regulation of milk secretion and quality particularly in the transition period from colostrum to milk in goats is limited^15,16^.

In order to assess physiological changes in transition milk of goats, changes in macromolecules (fat, protein, lactose etc.), some milk parameters (pH, electrical conductivity, freezing point, FFDM etc.) and fatty acid profiles of mammary secretion from parturition to 28th day in Damascus goats were determined in the present study. Furthermore, expression analysis of the genes on fatty acid profile, inflammation and oxidative stress related pathways in milk somatic cells during transition to milk were conducted. In addition, the correlations were revealed between genes in the mentioned pathways, MDA and SCC.

## Results

**Figure 1:**
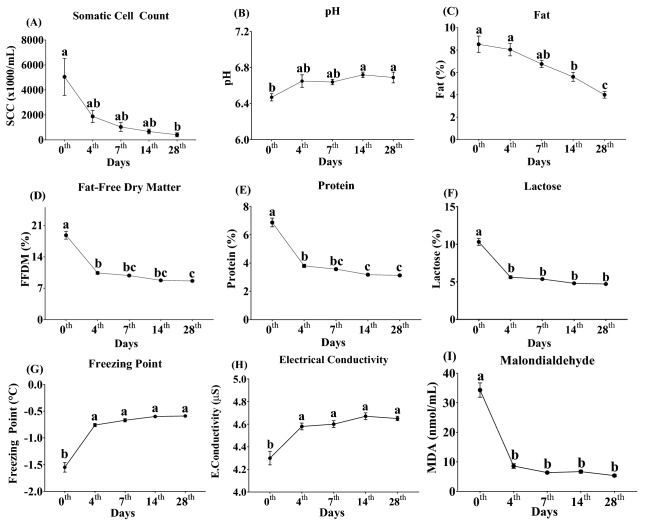
Somatic cell count, pH and compositional parameters on sampled days (Mean ± SEM); (**a**–**c**) Means followed by different letters differ significantly; SCC: Somatic cell count; FFDM: Fat-free dry matter; E. Conductivity: Electrical conductivity; MDA: Malondialdehyde.

The properties of colostrum or milk obtained have been presented in Fig. 1. The SCC was around 5 million on the 0th day, then it decreased dramatically on the 4th day and was around 1 million by the 7th day. Following the lactation, the SCC was around 400 × 10^3^/mL in milk (*P* < 0.01). While pH value was increased day by day, the levels of fat (%), FFDM (%), protein (%), and lactose (%) were decreased with the varying levels of significance (*P* < 0.001). On the other hand, the freezing point and electrical conductivity of the samples increased on the 4th day and remained at similar levels on the following days (*P* < 0.001). The level of MDA was highest with the 34.41 ± 2.43 nmol/mL values in the 0th day (*P* < 0.001). However, it was 3 times lower on the following days and there were no significant differences between these days.

The proportions of short and medium-chain fatty acids significantly changed with the continuation of lactation. While C4:0, C6:0, C8:0, C10:0, C15:1 fatty acids were at lowest level, they had gradually increased (*P* < 0.001). Moreover, a fluctuating change was observed in C12:0, C16:1, C17:0, C17:1, C18:2 *trans,* C18:2 *cis* fatty acid levels. On the other hand, C14:0 and C16:0 fatty acids decreased towards to 28th day. Moreover, C18:3 n3, C20:1, C20:2 n6, and C20:0 increased in 28th day, and C20:4 n6 was higher in 0th day than the other days (*P* < 0.001). Saturated fatty acids (SFA), monounsaturated fatty acids (MUFA) and unsaturated fatty acids (UFA) were similar in all sampled days. Furthermore, significant changes were determined in terms of polyunsaturated fatty acids (PUFA) of mammary secretion (*P* < 0.001). The n6 and n3 fatty acid levels of samples were highest on 28th day (*P* < 0.01; *P* < 0.001, respectively). However, on 28th day, n6/n3 ratio was significantly lower than those of other days (*P* < 0.01). Except for 0th day, odour index was similar on all days. The lowest odour index was found on 0th day (*P* < 0.001). In addition to nutritional value, atherogenic and thrombogenic indexes values were similar (Table 1).

**Table 1:** Fatty acids profile of milk samples in different stages of lactation; *: *P* < 0.05; **: *P* < 0.01; ***: *P* < 0.001; NS: Not significant; a, b, c, d: Means with different letters in rows differ significantly; OI (Odour index); NV (Nutritional value); AI (Atherogenic index); TI (Thrombogenic index) were calculated as follow: OI = (C4:0 + C6:0 + C8:0 + C10:0)^17–19^; NV = (C18:0 + C18:1)/C16:0; AI = (C12:0 + (4*C14:0) + C18:0)/ΣUFA^20^; TI = (C14:0 + C16:0 + C18:0)/((0.5*C18:1) + (0.5*ΣMUFA) + (0.5*Σn6) + (3*Σn3) + (Σn3/Σn6))^20^.

Parameters	0th day	4th day	7th day	14th day	28th day	*P*
C4:0	0.25 ± 0.03^d^	0.74 ± 0.07^c^	1.14 ± 0.09^ab^	0.92 ± 0.13^bc^	1.50 ± 0.11^a^	***
C6:0	0.59 ± 0.09^c^	1.38 ± 0.10^b^	1.90 ± 0.11^a^	1.73 ± 0.16^ab^	1.92 ± 0.14^a^	***
C8:0	0.83 ± 0.14^b^	1.78 ± 0.14^a^	2.12 ± 0.17^a^	2.21 ± 0.19^a^	2.08 ± 0.21^a^	***
C10:0	2.95 ± 0.43^b^	5.47 ± 0.4^a^	6.16 ± 0.41^a^	6.42 ± 0.52^a^	5.87 ± 0.64^a^	***
C12:0	1.79 ± 0.16^b^	2.47 ± 0.18^ab^	2.60 ± 0.18^a^	2.50 ± 0.18^ab^	2.19 ± 0.20^ab^	*
C14:0	8.70 ± 0.54^a^	7.58 ± 0.40^ab^	7.34 ± 0.38^ab^	7.13 ± 0.46^ab^	6.42 ± 0.41^b^	*
C14:1	0.08 ± 0.01	0.08 ± 0.01	0.07 ± 0.01	0.06 ± 0.01	0.06 ± 0.01	NS
C15:0	0.49 ± 0.02	0.49 ± 0.02	0.47 ± 0.03	0.46 ± 0.02	0.46 ± 0.02	NS
C15:1	0.20 ± 0.01^b^	0.18 ± 0.0^b^	0.20 ± 0.01^b^	0.22 ± 0.02^b^	0.28 ± 0.01^a^	***
C16:0	29.14 ± 0.97^a^	26.01 ± 0.59^b^	24.56 ± 0.56^bc^	24.41 ± 0.63^bc^	22.89 ± 0.70^c^	***
C16:1	1.17 ± 0.03^a^	0.98 ± 0.04^b^	0.79 ± 0.07^c^	0.83 ± 0.05^bc^	1.00 ± 0.02^ab^	***
C17:0	1.12 ± 0.05^ab^	0.97 ± 0.05^b^	0.98 ± 0.04^b^	0.99 ± 0.06^ab^	1.31 ± 0.17^a^	*
C17:1	0.55 ± 0.04^ab^	0.52 ± 0.05^b^	0.63 ± 0.06^ab^	0.58 ± 0.05^ab^	0.78 ± 0.09^a^	*
C18:0	19.29 ± 0.94	19.38 ± 1.18	20.08 ± 1.41	21.41 ± 1.52	18.61 ± 0.89	NS
C18:1	23.08 ± 0.76	22.83 ± 0.94	21.41 ± 0.92	20.41 ± 1.00	20.43 ± 0.85	NS
C18:2 *trans*	0.88 ± 0.12^ab^	0.66 ± 0.08^b^	0.57 ± 0.06^b^	0.69 ± 0.08^ab^	1.08 ± 0.19^a^	*
C18:2 *cis*	4.21 ± 0.22^ab^	3.85 ± 0.27^b^	4.69 ± 0.17^ab^	4.46 ± 0.26^ab^	5.30 ± 0.48^a^	*
C20:0	0.38 ± 0.04^b^	0.32 ± 0.03^b^	0.41 ± 0.04^b^	0.40 ± 0.04^b^	0.62 ± 0.07^a^	***
C18:3 n6	0.52 ± 0.05	0.52 ± 0.06	0.50 ± 0.04	0.47 ± 0.05	0.57 ± 0.06	NS
C18:3 n3	0.95 ± 0.18^b^	1.13 ± 0.19^b^	1.21 ± 0.12^b^	0.97 ± 0.09^b^	2.61 ± 0.48^a^	***
C20:1	0.65 ± 0.09^b^	0.49 ± 0.06^b^	0.56 ± 0.07^b^	0.61 ± 0.06^b^	1.12 ± 0.20^a^	***
C20:2 n6	0.33 ± 0.06^b^	0.27 ± 0.03^b^	0.28 ± 0.03^b^	0.43 ± 0.06^b^	0.76 ± 0.13^a^	***
C22:0	0.11 ± 0.02	0.12 ± 0.02	0.10 ± 0.01	0.12 ± 0.02	0.16 ± 0.03	NS
C20:3 n3	0.13 ± 0.02	0.11 ± 0.03	0.07 ± 0.01	0.09 ± 0.01	0.12 ± 0.02	NS
C20:4 n6	0.51 ± 0.04^a^	0.32 ± 0.02^b^	0.26 ± 0.02^b^	0.22 ± 0.02^b^	0.27 ± 0.04^b^	***
C22:2 n6	0.09 ± 0.01	0.10 ± 0.01	0.07 ± 0.01	0.09 ± 0.01	0.08 ± 0.01	NS
C20:5 n3	0.10 ± 0.01	0.09 ± 0.01	0.07 ± 0.01	0.08 ± 0.01	0.08 ± 0.01	NS
C24:1	0.88 ± 0.13	1.12 ± 0.32	0.72 ± 0.11	1.05 ± 0.20	1.40 ± 0.29	NS
C22:6 n3	0.03 ± 0.005	0.04 ± 0.003	0.03 ± 0.002	0.03 ± 0.003	0.04 ± 0.004	NS
SFA	65.64 ± 1.25	66.72 ± 1.30	67.87 ± 1.20	68.71 ± 1.24	64.00 ± 1.51	NS
MUFA	26.60 ± 0.85	26.19 ± 1.06	24.38 ± 1.01	23.76 ± 1.11	25.08 ± 0.95	NS
PUFA	7.76 ± 0.53^b^	7.09 ± 0.35^b^	7.75 ± 0.34^b^	7.53 ± 0.39^b^	10.92 ± 1.20^a^	***
UFA	34.36 ± 1.25	33.28 ± 1.30	32.13 ± 1.20	31.29 ± 1.24	36.00 ± 1.51	NS
n6	6.68 ± 0.40^ab^	5.84 ± 0.32^b^	6.44 ± 0.24^ab^	6.44 ± 0.35^ab^	8.19 ± 0.79^a^	***
n3	1.21 ± 0.20^b^	1.36 ± 0.20^b^	1.37 ± 0.13^b^	1.17 ± 0.09^b^	2.86 ± 0.50^a^	***
n6/n3	7.46 ± 0.8^a^	6.01 ± 0.68^a^	5.51 ± 0.52^ab^	6.23 ± 0.66^a^	3.61 ± 0.38^b^	***
OI	4.63 ± 0.67^b^	9.38 ± 0.69^a^	11.33 ± 0.68^a^	11.28 ± 0.92^a^	11.32 ± 1.00^a^	***
NV	1.49 ± 0.06	1.65 ± 0.07	1.71 ± 0.07	1.74 ± 0.08	1.73 ± 0.08	NS
AI	1.71 ± 0.13	1.66 ± 0.11	1.70 ± 0.12	1.75 ± 0.12	1.35 ± 0.09	NS
TI	1.50 ± 0.09	1.45 ± 0.08	1.50 ± 0.09	1.55 ± 0.09	1.27 ± 0.07	NS

**Figure 2:**
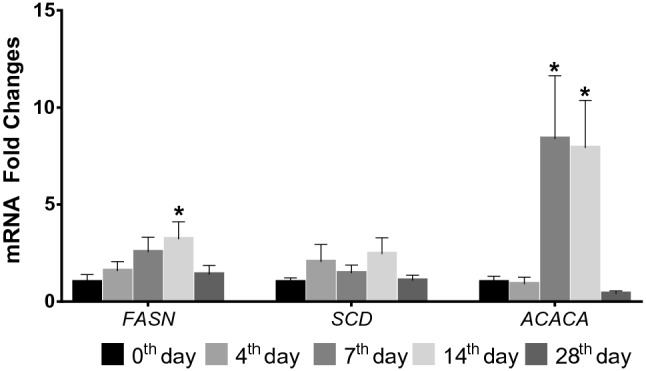
Expression levels of *FASN* (Fatty Acid Synthase), *SCD* (Stearoyl-CoA Desaturase), and *ACACA* (Acetyl-CoA Carboxylase Alpha) genes in milk somatic cells (Mean ± SEM); *: *P* < 0.05, Fold change differences in 4th, 7th, 14th and 28th days were compared to 0th day.

Expression patterns of the genes associated with fatty acid synthesis and secretion showed variable regulation. Compared to 0th day, *FASN* (Fatty Acid Synthase) was upregulated almost threefolds in 14th day (*P* < 0.05). While *SCD* (Stearoyl-CoA Desaturase) was similar, *ACACA* (Acetyl-CoA Carboxylase Alpha) was upregulated more than fivefolds in 7th and 14th days (*P* < 0.05) (Fig. 2).

**Figure 3:**
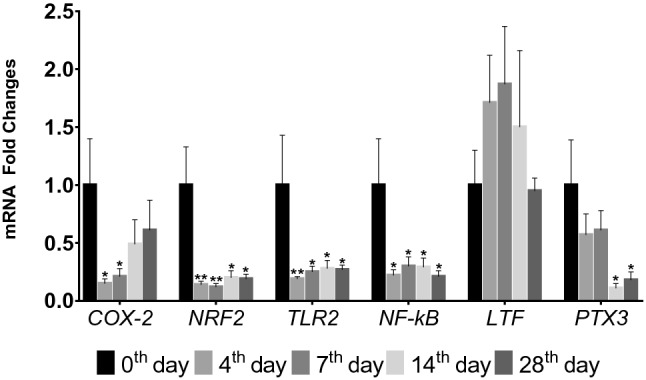
Expression levels of *COX-2 (*Cyclooxygenase-2*)*, *NRF2* (Nuclear Factor Erythroid 2-related Factor 2), *TLR2* (Toll-like Receptor 2), *NF-kB* (Nuclear Factor Kappa B), *LTF* (Lactoferrin), and *PTX3* (Pentraxin 3) genes in milk somatic cells (Mean ± SEM); *: *P* < 0.05; **: *P* < 0.01, Fold change differences in 4th, 7th, 14th and 28th days were compared to 0th day.

The marked downregulation was determined at oxidative stress and inflammation related genes. *COX-2* (Cyclooxygenase-2) was downregulated more than twofolds in 4th and 7th days (*P* < 0.05). On the other hand, *NRF2* (Nuclear Factor Erythroid 2-Related Factor 2) and *TLR2* (Toll-like Receptor 2) were approximately threefolds downregulated in all days with the different significances (*P* < 0.05 and *P* < 0.01). Therewithal, *NF-kB* (Nuclear Factor Kappa B) was downregulated on all days, while *PTX3* (Pentraxin 3) was downregulated close to fourfolds in 14th and 28th days (*P* < 0.05) (Fig. 3).

Positive and negative correlations with varying significance were detected between expression levels of related genes, SCC, and MDA (Table 2).

**Table 2:** Correlations between *FASN* (Fatty Acid Synthase), *SCD* (Stearoyl-CoA Desaturase; *ACACA* (Acetyl-CoA Carboxylase Alpha), *COX-2* (Cyclooxygenase-2), *TLR2* (Toll-like Receptor 2), *LTF* (Lactoferrin), *PTX3* (Pentraxin 3), *NF-kB* (Nuclear Factor Kappa B), *NRF2* (Nuclear Factor Erythroid 2-related Factor 2), SCC (Somatic Cell Count) and MDA (Malondialdehyde); *: *P* < 0.05; **: *P* < 0.01; ***: *P* < 0.001; -: Not significant.

Parameters	*FASN*	*SCD*	*ACACA*	*COX-2*	*TLR2*	*LTF*	*PTX3*	*NF-kB*	*NRF2*	SCC
*SCD*	0.589***									
*ACACA*	0.365**	0.249*								
*COX-2*	–	–	–							
*TLR2*	− 0.221*	–	–	0.359**						
*LTF*	0.393***	0.485***	0.280*	–	–					
*PTX3*	− 0.275*	–	–	–	0.405***	–				
*NF-kB*	–	–	–	0.517***	0.713***	–	0.372**			
*NRF2*	–	–	–	0.479***	0.584***	–	–	0.611***		
SCC	− 0.407***	− 0.244*	–	–	0.400***	− 0.318**	0.475***	0.412***	0.237*	
MDA	–	–	–	0.273*	0.453***	–	0.412***	0.349**	0.580***	0.438***

## Discussion

Similar to the study conducted in goats by Moreno-Indias et al.^8^, SCC, which was initially about 5000 × 10^3^/mL in colostrum, tended to gradually decrease during the transition to milk in this study. Sanches-Macias et al.^9^ reported that from the 0th day to the 4th day, SCC had decreased from approximately 9000 × 10^3^/mL to 3000 × 10^3^/mL levels, and after the 15th day it had remained below 1000 × 10^3^/mL as this studies results. High SCC in colostrum was thought to be physiological and the possible cause, as reported in other studies, might be due to the leaky tight junctions between mammary epithelial cells^9,21^. In addition, breed type of goat was thought to be an important factor for differences in terms of SCC during transition colostrum to mature milk^22–24^. While pH was lower on 0th day, it gradually increased during transition similar to other studies^7,25,26^. It was reported that lower pH might be related to mastitis and bacterial contamination in milk^9^, however, the colostrum and transition milk have also physiologically lower pH values than milk according to our findings. It was possible to evaluate this situation as one of the physiological self-protection mechanisms of the mammary gland during the transition period.

The possible reason of higher lactose content in the initial samples is that the lactose is a source of energy that may be digested faster than fat and required for newborn^8^. While most of the milk quality parameter has dramatically decreased during transition, freezing point and electrical conductivity has increased since 4th day of lactation. Expectative reason of increases on these parameters are decreased percentages of parameters such as fat, protein, FFDM of secretion in addition to other compounds as minerals. Also, it has been reported that genetic background of breeds affected the composition of colostrum and milk in goats^10^.

MDA is a primary biomarker for determination the lipid peroxidation and oxidative stress in tissues. Moreover, it has been reported that lactation period strongly related with milk MDA levels and it has higher values in early lactation stage^27^. While significantly higher values have been found in colostrum, MDA has dramatically decreased with the continuation of lactation. In a study, it has been reported that composition and yield of milk is significantly correlated with MDA levels in cow milk^27^. According to correlation results in our study, high SCC is also associated with increased MDA levels in mammary secretion. Although milk MDA levels are understandably dependent on milk composition, more exploration at the molecular levels is needed to elucidate the mechanism of MDA in colostrum and milk.

It is essential to explore the milk fatty acid profile at different lactation stages to achieve the optimal benefits from milk^28,29^. It has been determined that short-chain fatty acids responsible for the odour index in milk has tended to increase during the transition to mature milk. Beside short-chain fatty acids, medium-chain fatty acids have also increased during transition. It has reported that medium-chain fatty acids (C8:0, C10:0 and C12:0) in milk have antimicrobial effects^30^. Goat kids are susceptible to infections and therefore medium-chain fatty acids in secretion may have increased for the protective effects on young goats^28^. While short and medium-chain fatty acids and some of the long-chain fatty acids have gradually increased in the transition from colostrum to milk, some of the long-chain fatty acids have stayed stable from 0 to 28th day. It has been thought that the likely cause of these results is unchanged ration ingredients of animals. This is because of the fact that it is known that composition of ration has major effect on the fatty acid profile of goat milk^31^.

PUFA, odour index, n6, and n3 have increased with the continuation of lactation, while n6/n3 ratio has decreased. Hence, milk has become better quality with the transition period^32^. PUFA has been reported to decrease towards the end of the lactation in different goat breeds^33^. However, it has increased in the transition processes from colostrum to mature milk in this study. Although it has known that nutrition has important effects on milk fatty acid profile, studies show that the lactation period is another major point on this parameter^34,35^. While there have been controversial reports about fatty acid profile of milk in different lactation stages^33,36^, it has been showed up fatty acid profile of goat milk is one of the most variable components among the lactation period, together with milk yield and composition^35^.

The molecular regulation of lipid metabolism remains largely unknown in ruminants. Fatty acid biosynthesis is an intriguingly complex biological process and *FASN*, *SCD,* and *ACACA* are largely effective genes in lipid biosynthesis. *FASN,* one of the highly expressed gene in goat colostrum, has been reported required for the maintenance of lactation^34,37^. Therewithal, *FASN* is mostly responsible to synthesis of short and medium-chain fatty acids together with *ACACA*^34^. It has been reported that inhibition of *FASN* led to reduce of medium-chain fatty acids and downregulation of *ACACA* in mammary gland of goat^38^. In addition, *FASN* in goat mammary gland might synthesize all three fatty acid forms (short, medium and long-chain fatty acids)^38^. It has been reported that *FASN*, *ACACA*, and *SCD* regulated together from pregnancy to end of the lactation in bovine mammary gland^39^. In recent studies, *FASN*, *SCD* and *ACACA* genes have been mentioned that they might be candidate genes for qualified animal product from the point of fatty acid profile^40–43^. While some researches have studied on expression patterns of *FASN*, *SCD* and *ACACA* genes in goats^37,40,43^, to our knowledge there is no report about the activities of these genes in colostrum and transitional milk somatic cells of goats. It has been clearly understood that despite the differences in upregulation levels, regulation of fatty acid synthesis in goat colostrum and early milk requires coordinately expression of mentioned genes.

Antioxidant status of colostrum is important for maternal physiology and offspring health^44^. *COX-2* and *NRF2* are the most related genes with the oxidative status of tissues and biological liquids and have tended to be upregulation in response to immunological activity^45,46^. It has been reported that *NRF2* increases by triggering *TLR2* in leukocytes and oxidative stress may decrease by overexpressing *TLR2* in goats^47^. In inflammation status, oxidative stress initiates the inflammatory response by activating *NF-kB* and increases the expression levels of target genes such as *COX-2*. It has been reported that response to oxidative stress is formed by cross-interaction between *NRF2* and *NF-kB*^48^.

*PTX3*, which expression level has decreased with the transition from colostrum to mature milk, is thought to be regulated for both kid and maternal health. *PTX3* has known to be a gene regulated by the molecular mechanisms of innate resistance to respond to infectious agents^13,49^. There has been limited knowledge about the activity and expression patterns of *PTX3* in healthy goats, while one of the most expression of *PTX3* has been reported is mammary gland^50^. It has been stated that *PTX3* gene might be upregulated by inflammation in mammary tissue and its activity might be used an early marker of mastitis^50,51^. It has been advocated that whether there is a correlation between *PTX3* and SCC should be investigated in goat milk^50^. The results obtained from our study show that in addition to relations with the oxidative and inflammatory genes such as *TLR2* and *NF-kB*, there is crucial correlation between *PTX3* and SCC in early lactation of goats.

In addition to the antimicrobial activities of medium-length fatty acids synthesized by the activities of *FASN*, *SCD* and *ACACA*, *LTF* has also antimicrobial activity in mammary gland^52^. In fact, in recent studies, researchers have mentioned that the combination effects of *LTF* and fatty acids regulated from these genes activities are more effective against to microbial agents^53,54^. However, what is known about the relation between fatty acid synthesis and *LTF* activity is quiet limited. Therefore, more research is needed on direct and indirect relations between *LTF* and fatty acid biosynthesis in mammary gland.

Indicator of oxidative stress and cytokines have known transferred from mother with colostrum and milk, may affecting the health of offspring^55^. On a study it has reported that it is appropriate to weaning of lambs at 28 days^56^. In addition to health of kids and goats, transition and mature milk should be investigated at the molecular levels for milk quality parameters.

To our knowledge, this is the first study to deeply explore the changes at the molecular levels (mRNA expressions in inflammation, oxidative stress and fatty acid synthesis related pathways) of mammary secretion of goats from birth to 28th day of lactation. In transition process from colostrum to mature milk, fatty acid biosynthesis is substantially regulated by the activities of *FASN*, *SCD* and *ACACA* in somatic cells. In addition, mammary physiology of goats strongly related with co-regulation of *COX-2*, *NRF2*, *TLR2*, *NF-kB*, and *PTX3* genes in first 28 days of lactation. It is possible to deduce that indirect interactions of *LTF* and fatty acid synthesis related genes (*FASN*, *SCD*, and *ACACA*) are involved in the regulation of antimicrobial activity of mammary gland. In conclusion, variable interactions between genes have showed that the regulation of goat milk are intriguingly reorganized in transition process. The additive effects of studied genes have significant roles on properties of colostrum, transition and mature milk in goats. To elucidate the underlying molecular mechanism of the goat mammary secretion, more comprehensive study is needed in this field.

## Methods

### Materials, design of the study, samples collection and measuring parameters

This research was conducted with Damascus goats aged 3–4 years (40.54 ± 0.71 months old) in a private goat enterprise located at 36° 21′ 52.6′′ N and 36° 15′ 14.6′′ E at an altitude of 82 m above sea level in the Eastern Mediterranean region of Turkey (Hatay Province). Healthy 24 multiparous goats were randomly selected from 200 goats flock. All parturitions were completed in the first week of February in 2020 (each goat gave singleton birth). The goats were in their 2nd–3rd lactation period (2.58 ± 0.10). Goats consumed 1.2 kg/goat concentrated feed and 1.0 kg/goat wheat straw on a daily basis (Table 3). Animals were fed twice daily (07:00 and 16:00 h) and housed in a pen (2 m^2^ of floor space per animal). Following parturitions, goats were hand-milked twice daily and approximately 150 mL colostrum or morning milk samples were collected to nuclease free falcon tubes on the 0th, 4th, 7th, 14th, and 28th days. Prior to sampling, udders and teats of goats were cleaned with sterile cotton gauzes. Lactation period was continued approximately 210–220 days.

**Table 3:** Chemical and physical composition of concentrate feeds; *: Per 1.5 kg premix contains 15 000 000 IU Vit A, 3 000 000 IU Vit D3, 50 000 IU Vit E, 50 g manganese, 50 g ferrous, 50 g zinc, 10 g copper, 0.8 g iodine, 0.2 g cobalt, 0.3 g selenium.

Items contents	Proportions (%)
Wheat	19.50
Maize barn	20.60
Corn	18.50
Sunflower meal	15.50
Cottonseed meal	10.00
Barley	7.50
Wheat barn	2.30
Molasses	5.00
Marble powdered (%38 Ca^++^)	0.30
NaCl	0.70
Premix*	0.10
Total	100.00
Dry matter	88.91
Crude ash	5.96
Ether extract	2.58
Crude protein	16.51
Total metabolic energy (kcal/kg)	2649.28

Samples were transported to the laboratory at 4 °C in 30 min. Approximately quarter of each sample was used for determination of SCC (Lactoscan SCC 6010, BULGARIA) and pH (Hanna pH meter, HI83141, USA) values. Fat, FFDM, protein, lactose, freezing point, and electrical conductivity parameters were measured with milk analyzer (Milkotester Master Classic LM2-P1, BULGARIA). In addition, the levels of MDA were determined with UV-Spectrophotometer at 532 nm wavelength^57^. All parameters were measured within 2 h of milking in two replicates and mean values were recorded.

### Cream layer and somatic cells collection

Approximately 50 mL of milk sample were centrifuged at + 4 °C at 1800 × *g* for 15 min (for colostrum samples, 25 ml of colostrum were completed to 50 ml with the same volume of PBS and homogenized). Thereafter the samples were kept for about 15 min at − 20 °C. After the cream layer was collected and stored − 20 °C for fatty acid analyzes, the supernatant phase was poured out and PBS was added to the bottom cell pellet and homogenized. Centrifugation was repeated at + 4 °C at 1800 × *g* for 15 min. Supernatant phases of samples were discarded and approximately 1 mL TRIzol Reagent (Sigma-Aldrich, USA) was added to cell pellets and homogenized by pipetting. Following the homogenization, samples were stored − 86 °C until RNA isolation.

### Fatty acid analyzes

For fatty acid profiles of samples, 500 µL cream was used from each sample. Samples were homogenized with 2 mL of 2 N methanolic KOH for 4 min at room temperature. Then 4 ml of n-Heptane (Merck, USA) was added to samples and kept for 2 min at room temperature. Following the centrifugation at 200 × *g* for 5 min, the aqueous phases of samples containing methyl esters were transferred to 1.5 mL vials. Fatty acids of samples were determined by Gas Chromatography equipped with flame ionization detector (Shimadzu GC-2025, Japan), auto-injector (Shimadzu AOC-20i, JAPAN) and Restek Rt-2560 column (100 m length, 0.25 mm ID × 0.20 µm). Temperatures of injector and detector were both kept at 250 °C. Hydrogen was used as carrier gas and the gas flow was 1.20 mL/min. Injection mode was split mode with split ratio of 1:50 and total injection volume was 1 μL. Injector was rinsed with n-Heptane, three times pre-run and six times post-run. Temperature gradient program was used. The initial oven temperature was 100 °C (hold for 2 min) and it was then increased by 4 °C/min until 250 °C (hold for 15 min). The run was 54.50 min. For the verification of fatty acids, the determined sample peaks retention times were compared with that of internal standard (FAME Mix, Restek, USA). In addition to nutrition value (NV), odour index (OI), atherogenic index (AI), and thrombogenic index (TI) were calculated as below:

NV = (C18:0 + C18:1)/C16:0.

OI = (C4:0 + C6:0 + C8:0 + C10:0)^17–19^;

AI = (C12:0 + (4*C14:0) + C18:0)/ΣUFA^20^;

TI = (C14:0 + C16:0 + C18:0)/((0.5*C18:1) + (0.5*ΣMUFA) + (0.5*Σn6) + (3*Σn3) + (Σn3/Σn6))^20^.

### Total RNA isolation, genomic DNA digestion and cDNA synthesis

Total RNA was isolated from the somatic cells by standard TRIzol method^58^. According to protocol, 250 µL chloroform was added to cell suspensions homogenized in TRIzol Reagent and gently mixed. After waiting 10 min at room temperature, samples were centrifuged at + 4 °C at 12,000 × *g* for 15 min. Aqua phases of samples were collected to new sterile-nuclease free centrifuge tubes and isopropyl alcohol was added as much as half of the TRIzol Reagent added in the samples. The samples were briefly mixed and stored up at room temperature for 10 min. For precipitation RNA, samples were centrifuged at 4 °C at 12,000 × *g* for 10 min. Supernatants of samples were discarded following the centrifuge and 1 mL 70% ethyl alcohol was added to sample. The samples added 70% ethyl alcohol were centrifuged at 4 °C at 7500 × *g* for 5 min. This step was performed twice. Samples were centrifuged for the last time with 1 mL 96% ethyl alcohol at similar conditions. Finally, RNA pellets were kept at room temperature for 10 min then dissolved with 30–100 µL nuclease-free water. Purity (A_260/280_ = 1.85 ± 0.01) and concentration of RNA (233.86 ± 13.42 ng/µL) were controlled by nucleic acid spectrophotometer (Merinton SMA-1000 UV Spectrophotometer, CHINA). In addition, quality of RNA was checked by evaluated 28S and 18S rRNA bands with 1% agarose gel electrophoresis (100 V and 25 min).

DNA digestion protocol was carried out to samples for eliminating possible genomic DNA contamination (DNase I, RNase free, Thermo Fisher Scientific, USA). Total RNA was then converted to cDNA with using cDNA synthesis kit (RevertAid First Strand cDNA Synthesis Kit, Thermo Fisher Scientific, USA). Protocol of thermal cycler (Bio-Rad T100, USA) was as follows: Samples were kept at 25 °C for 10 min, subsequently at 37 °C for  20 min, and then at 85 °C for 5 min. Following the reaction, sample was completed to 150 µL and kept at − 20 °C until gene expression analyzes.

### Real-Time qPCR application

Amplifications of *FASN, SCD, ACACA, COX-2, NRF2, TLR2, NF-kB, LTF,* and *PTX3* were performed using 10 µL of each cDNA samples in RT-qPCR (Bio-Rad CFX-96 Touch Real time PCR, USA). SYBR Green I dye containing kit (Power SYBR Green PCR Master, Thermo Fisher Scientific, USA) was used for amplification and each sample was studied as duplicated. The reaction was arranged 10 min at 95 °C, followed by 15 s at 95 °C, 60 s at 60 °C, and 40 cycles in RT-qPCR. On the other hand, *ACTB* and *G6PD* housekeeping genes were used as internal control. Forward and reverse sequences of primers were shown in Table 4.

**Table 4:** Forward and reverse sequences of primers amplified genes; *: Designed by authors, P.L.: Product Length; FASN: Fatty Acids Synthase; SCD: Stearoyl-CoA Desaturase; ACACA: Acetyl-CoA Carboxylase Alpha; COX-2: Cyclooxygenase-2; TLR2: Toll-like Receptor 2; NF-kB: Nuclear factor Kappa B; LTF: Lactoferrin; PTX3: Pentraxin 3; NRF2: Nuclear Factor Erythroid 2-related Factor 2; ACTB: Actin-beta; G6PD: Glucose-6-phosphate Dehydrogenase.

Genes	Accession no	Forward and reverse sequences of primers	P.L	Ref
*FASN*	NM_001285629.1	F: 5′-GCACACAATATGGACCCCCA-3′ R: 5′-CATGCTGTAGCCTACGAGGG-3′	183	*
*SCD*	NM_001285619.1	F: 5′-ATCGCCCTTACGACAAGACC-3′ R: 5′-CATAAGCCAGACCGATGGCA-3′	186	*
*ACACA*	XM_018064168.1	F: 5′-GCCTGCCCGAGTTTTGAGTG-3′ R: 5′-CGCACTCTGGAGCGGATAAA-3′	105	*
*COX-2*	XM_018060731.1	F: 5′-GTAGGCCAGGAGGTCTTTGG-3′ R: 5′- GCCTGCTTGTCTGGAACAAC-3′	142	*
*NRF2*	NM_001314327.1	F: 5′-CTGTTCTCTGCTGTCAAGGG-3’ R: 5′-AACTCGCCGGTCTCTTCATC-3’	222	*
*TLR2*	NM_001285603.1	F: 5′-TGCTGTGCCCTCTTCCTGTT-3′ R: 5′-GGGACGAAGTCTCGCTTATGAA-3′	260	^47^
*NF-kB*	XM_018049265.1	F: 5′-TGGGGATACTGAACAACGCC-3′ R: 5′-ATCTGTCTCAGGGCCTCCAT-3′	115	*
*LTF*	NM_001285548.1	F: 5′-CAAGTGTGTGCCCAACTCTA-3′ R: 5′-GCTCTCTCCATTCGTGTTCTC-3′	105	^19^
*PTX3*	XM_018048475.1	F: 5′-CCTGCATTTGGGTCAAAGCC-3′ R: 5′-AATCACAGCATCAGCGACCA-3′	186	*
*ACTB*	XM_018039831.1	F: 5′-TGGATCGAGCATCCCCAAAG-3′ R: 5′-ACTGGCCCCTTCTCCTTAGA-3′	169	*
*G6PD*	XM_018044338.1	F: 5′-TGACCTATGGCAACCGATACAA-3′ F: 5′-CCGCAAAAGACATCCAGGAT-3′	76	^59^

### Statistical analysis

Descriptive statistics for each variable were calculated and presented as “Mean ± Standard Error of Mean”. The Pearson correlation coefficient was used to determine the correlation between gene expression levels, somatic cell count and MDA levels. To determine the effect of time of sampling on milk quality and milk fatty acid parameters, linear mixed model was used. The following model with repeated measures design: ${Y}_{ijk}= \mu +{T}_{j}+ {e}_{ijk}$ . Where, ${Y}_{ijk}$, dependent variable; $\mu$, overall mean; ${T}_{j}$, effect of time of sampling (j = Day of parturition, after parturition 4, 7, 14 and 28 d) and ${e}_{ijk}$, residual error. Animals were assessed as a random effect, while the time of sampling was assessed as fixed effect. When a significant difference was revealed, any significant terms was compared by simple effect analysis with Bonferroni adjustment. *P* < 0.05 was considered as significant in all analyses. All data were analyzed using IBM SPSS Statistics software (Version 23.0)^60^.

Expression levels of genes were calculated by the 2^−ΔΔCt^ method and geometric mean of reference genes Ct values was used for gene expression analyzes^61^. The results were determined with comparing to 0th day and presented as fold changes.

### Ethical statement

All methods and procedures strictly complied with the “Regulation on the Studying Procedures and Principles of Animal Experiments of Ethics Committees” of Ministry of Agriculture and Forestry (2014, Republic of Turkey) and regulations of the Animal Experiments Local Ethics Committees of Hatay Mustafa Kemal University. In addition, the approval of the farm owner was obtained for the study.
